# The Negro

**Published:** 1900-05-05

**Authors:** 


### THE NEGRO.

Events in South Africa tend to give prominence to the negro question, which is not unlikely to give rise to serious differences of opinion when the task of settling affairs in that part of the world comes to be undertaken. American experience does not seem to be an altogether happy one, especially in regard to the degeneracy of the race under the influence of civilisation without control. Dr. Paul B. Barringer, of the University of Virginia, who recently gave an address on the subject before the Tri-State Association of Virginia and the Carolinas, after referring to the cannibalistic propensities of the West Coast African negro of the present day, speaks of the American negro as follows: "Thirty-five years have passed since the negro changed from the condition of a slave to that of a freed-man. In every part of the south, it is the opinion of every man of unbiassed mind that the second generation is infinitely worse than the first. . . . The young negro of the South, except where descended from parents of exceptional character and worth, is reverting through hereditary forces to savagery. . . . Everything points to the fact that the phylogeny of the negro is carrying him back to barbarism; that the temporary elevation produced by the discipline of slavery is not being maintained by the efforts we have made at common-school education in the hands of his own race, and that we must at once, if we would save the negro and the South, try something else." The *Medical Record*, in reporting the above remarks, adds that it has many times called attention to the physical deterioration of the coloured race on the American continent, clearly owing to the insanitary conditions in which its members live. The matter is, it says, of the most serious importance, and yearly assumes a more ominous aspect.

